# Ampicillin and Gentamicin Are a Useful First-line Combination for the Management of Sepsis in Under-five Children at an Urban Hospital in Bangladesh

**DOI:** 10.3329/jhpn.v30i4.13418

**Published:** 2012-12

**Authors:** Samira Bibi, Mohammod Jobayer Chisti, Farhana Akram, Mark A.C. Pietroni

**Affiliations:** ^1^Pharmacy Department, East West University, Dhaka, Bangladesh; ^2^Dhaka Hospital and Centre for Nutrition and Food Security (CNFS), icddr,b, GPO Box 128, Dhaka 1000, Bangladesh; ^3^Centre for Nutrition and Food Security (CNFS), icddr,b, GPO Box 128, Dhaka 1000, Bangladesh

**Keywords:** Antibiotics, Children, Diarrhoea, Infant, Sepsis, Bangladesh

## Abstract

The study evaluated the commonly-used drugs for the management of sepsis and their outcome among under-five children. We evaluated the hospital-records of all paediatric sepsis patients (n= 183) in the intensive care unit (ICU) and longer-stay unit (LSU) of the Dhaka Hospital of icddr,b. These records were collected from the hospital management system (SHEBA) during November 2009 to October 2010. A total of 183 under-five children with clinical sepsis were found during the study period, and 14 (8%) of them were neonates. One hundred and eighty-one patients had received a combination of injection ampicilin and injection gentamicin, and two patients had received the combination of injection ceftriaxone and injection gentamicin. Only 46 (25%) patients required a change of antibiotics to the combination of intravenous ceftriaxone plus gentamicin after non-response of injection ampicilin and injection gentamicin combination; 7/181 (4%) patients died who received injection ampicilin and injection gentamicin whereas none died among the other two patients who received injection ceftriaxone and injection gentamicin (p=1.00). The combination of injection ampicilin and injection gentamicin as the first-line antibiotics for the management of sepsis in children even beyond the neonatal age is very effective, resulting in lower mortality.

## INTRODUCTION

Sepsis remains a significant cause of morbidity as well as mortality in the paediatric population (1-6). Most of these deaths occur in developing countries (6-8). Death rate is even higher when children with sepsis also have diarrhoea (9). Appropriate antibiotic therapy has been shown to be an important predictor of outcome in sepsis, decreasing morbidity and mortality rates among patients in the intensive care unit (10,11). In 2000, Ibrahim *et al.* conducted a prospective cohort study where they found that the inpatient mortality rate reduced from 61.9% for patients with inadequate treatment to 28.4% for patients receiving appropriate therapy (12). In 2004, MacArthur and colleagues found that adequate and early empirical antibiotic treatment was associated with a 10% decrease in the overall crude mortality rate from 43% for patients receiving inadequate antibiotic treatment to 33% in patients receiving adequate antibiotic support (13). Combination of injection ampicilin and injection gentamicin is the first choice in the neonatal age-group for the management of sepsis (14-19). However, data on the use of the combination of injection ampicilin and injection gentamicin in under-five children, beyond the neonatal age, with sepsis and diarrhoea are very limited. Dhaka Hospital of icddr,b treats a good number of children with sepsis (20). The aim of this study was to evaluate the commonly-used drugs for the management of sepsis among the paediatric patients and also to reveal the outcome of the patients receiving antibiotics.

## MATERIALS AND METHODS

### Study design

This study involves a prospective analysis of retrospective data on paediatric sepsis patients, collected from SHEBA, an online database of the Dhaka Hospital of icddr,b.

### Study population and site

We reviewed the retrospective data on the hospitalized children with sepsis. For this study, we prepared a questionnaire based on our topic and then extracted relevant data on all ICU and LSU paediatric sepsis patients (n=183) from November 2009 to October 2010 with the help of the analyst programmer of SHEBA at icddr,b. We have evaluated the common antibiotics used for the management of sepsis within this period. Every year, the Dhaka Hospital of icddr,b deals with 110,000 patients, and most of the patients have diarrhoea, with or without other complications (20). The Hospital also conducts research on various infectious diseases but mostly on diarrhoeal patients. After arrival, nurses record the medical history of patients and make quick assessment, focusing on the severity of disease as well as other complications. The targeted population for our study was the paediatric sepsis patients, and majority of the patients came from the poor socioeconomic background. Patients were discharged with oral antibiotics after complete recovery from the clinical signs of sepsis.

### Inclusion criteria of the cases

The study included under-five diarrhoeal children of both sexes with diagnosed sepsis.

### Definition of sepsis

Sepsis is defined as presence of any two of the following: tachypnoea, tachycardia, thermo-instability (hypo or hyperthermia), and abnormal WBC count (>11000/cc or, <4000/cc or, band and neutrophil ration ≥0.1) plus hypotension in the absence of clinical dehydration or after correction of dehydration (21).

### Ethical issues

Our research did not involve any interview of the patient or caregiver, and it was solely a chart analysis. The data were anonymized before being received by the researchers.

### Statistical analysis

The relevant data were entered into personal computer, using SPSS for Windows (version 17.0, SPSS Inc.) and then analyzed for parameters, including neonatal age, antibiotics, and outcome of the patients.

## RESULTS

There were 183 children with clinical sepsis during the study period, and 14 (8%) of them were neonates, 121 (66%) infants (1 month to <12 months), and the rest 48 (26%) were older (12 months to 59 months); 70 (38%) had received oral medication at home prior to injectable antibiotics on admission in the hospital; 181 patients had received combination of injection ampicilin and injection gentamicin; and two patients had received the combination of injection ceftriaxone and injection gentamicin as the first-line antibiotics. The median (inter-quartile range) duration of hospital stay for the patients was 6.7 (4.7-11.2) days; 7 patients died who received injection ampicilin and injection gentamicin whereas none died among the other two patients who received injection ceftriaxone and injection gentamicin combination (p=1.00) as the first-line antibiotics. Overall, 46 (25%) patients received second-line therapy (intravenous ceftriaxone plus gentamicin) after deterioration in the first-line therapy (combination of injection ampicilin and injection gentamicin). All patients who died received second-line therapy due to clinical deterioration. Among the patients who died, 2 had the growth of coagulase-negative staphylococcus (CNS) in blood and were sensitive to ongoing second-line antibiotics; 5/7 (71%) had septic shock (including inotrope resistant septic shock and multi-organ dysfunction syndrome in 2 cases and severe pneumonia in 2 cases) and 1 each had acute respiratory distress syndrome (ARDS) and severe acute malnutrition (SAM). Among the survivors, 11/174 (6%) had invasive bacteraemia due to *Streptococcus* species (three), *Streptococcus pneumonae* (one), *Staphylococcus aureus* (one), *Pseudomonas* species (two), *Klebsieilla* species (one), *Salmonella* Typhi (one), *Acinetobacter* species (one), and *Moraxella* species (one).

## DISCUSSION

Our analysis demonstrated the efficacy of commonly-used antibiotic therapy for the treatment of paediatric sepsis. We observed that 99% of under-five children with clinical sepsis received injection ampicilin and injection gentamicin as the first-line antibiotics, with an excellent survival rate of 96% (case-fatality rate 4%). Ampicillin and gentamicin are usually effective against all the bacterial agents causing community-acquired sepsis in neonates as this combination has traditionally been considered to have activity against both Gram-positive and Gram-negative organisms in the neonatal period (22). However, our data suggest that this combination may also be used in the initial therapy for clinical sepsis in under-five children. This is a notable finding of this study. The icddr,b hospital has a scrupulous adherence to a standard treatment protocol for fluid therapy, oxygen, and other supportive care with continuous monitoring and excellent follow-up. This might have an additional impact on the excellent survival with the first-line antibiotics.

The combination of ceftriaxone and gentamicin was used only for 2 patients as the first-line therapy among 183 clinically-diagnosed sepsis patients. In some centres, third-generation cephalosporins, in combination with gentamicin, have been used in the initial therapy for neonatal sepsis (23). Third-generation cephalosporins, such as ceftriaxone, have a broad-spectrum activity and further-increased activity against Gram-negative organisms (24-26). These may be particularly useful in treating Gram-negative bacteria causing severe forms of sepsis where a combination of injection ampicilin and injection gentamicin is ineffective. A combination of intravenous ceftriaxone and gentamicin should be preserved as the second-line therapy, if the first-line therapy (injection ampicilin and injection gentamicin) fails (on the basis of no clinical improvement 48 hours after initiation of therapy or clinical deterioration within 24 hours after initiation). We had to do this in only 25% of our study children. This indicates that 75% of the patients responded to the combination of injection ampicilin and injection gentamicin, which is a great achievement. WHO has also recommended this combination therapy as the treatment of choice for children with clinical sepsis at inpatient facilities (27). Although the recommended first-line treatment is the combination of injection ampicilin and injection gentamicin for all government medical college hospitals and district hospitals in Bangladesh, the physicians are often dubious of the effectiveness of this combination therapy and often rely on third-generation cephalosporins due to their unsubstantiated worry. The observed fatality among the patients who received the first-line combination therapy of injection ampicilin and injection gentamicin was not significant, and all of them had received injectable third-generation cephalosporin immediately after non-response of the first-line antibiotics. However, all the patients who deteriorated and died had serious consequences and co-morbidity, such as irreversible septic shock or ARDS or severe pneumonia or severe malnutrition. Case-fatality rate in such children is very high even in developed countries (20,28). Isolation of CNS from the blood might be due to contamination of the blood sample. On the other hand, 6% of the surviving patients who received injection ampicilin and injection gentamicin as the first-line antibiotics had invasive bacteraemia. This underscores the importance of the use of injection ampicilin and injection gentamicin combination as the first-line antibiotics in under-five children, beyond neonatal period, with sepsis with a scrupulous adherence to appropriate monitoring and follow-up. This combination therapy is very cheap and can be used at resource-limited settings, especially at district-level hospitals and upazila health complexes in Bangladesh.

### Conclusions

Our data suggest that the use of a combination of injection ampicilin and injection gentamicin for the management of clinical sepsis in children even beyond the neonatal age is very effective, and the mortality is low. Thus, this cheap therapy may be used as the first-line treatment of under-five children with clinical sepsis even beyond the neonatal age in all government medical college hospitals and public hospitals, including upazila health complexes. However, further research with a larger sample should be conducted to consolidate our observation.

## ACKNOWLEDGEMENTS

We gratefully acknowledge SHEBA personnel at icddr,b for their relentless support and help.
